# Elevation of artemisinin content by co-transformation of artemisinin biosynthetic pathway genes and trichome-specific transcription factors in *Artemisia annua*

**DOI:** 10.3389/fpls.2023.1118082

**Published:** 2023-02-21

**Authors:** Danial Hassani, Ayat Taheri, Xueqing Fu, Wei Qin, Liu Hang, Yanan Ma, Kexuan Tang

**Affiliations:** ^1^ Frontiers Science Center for Transformative Molecules, Joint International Research Laboratory of Metabolic and Developmental Sciences, Plant Biotechnology Research Center, Fudan-SJTU-Nottingham Plant Biotechnology R&D Center, School of Agriculture and Biology, Shanghai Jiao Tong University, Shanghai, China; ^2^ School of Life Sciences, East China Normal University, Shanghai, China

**Keywords:** artemisinin, co-expression, transcription factor, transformation, metabolic engineering

## Abstract

Artemisinin, derived from *Artemisia annua*, is currently used as the first-line treatment for malaria. However, wild-type plants have a low artemisinin biosynthesis rate. Although yeast engineering and plant synthetic biology have shown promising results, plant genetic engineering is considered the most feasible strategy, but it is also constrained by the stability of progeny development. Here we constructed three independent unique overexpressing vectors harboring three mainstream artemisinin biosynthesis enzymes *HMGR, FPS, and DBR2*, as well as two trichomes-specific transcription factors *AaHD1* and *AaORA.* The simultaneous co-transformation of these vectors by *Agrobacterium* resulted in the successful increase of the artemisinin content in T0 transgenic lines by up to 3.2-fold (2.72%) leaf dry weight compared to the control plants. We also investigated the stability of transformation in progeny T1 lines. The results indicated that the transgenic genes were successfully integrated, maintained, and overexpressed in some of the T1 progeny plants’ genomes, potentially increasing the artemisinin content by up to 2.2-fold (2.51%) leaf dry weight. These results indicated that the co-overexpression of multiple enzymatic genes and transcription factors *via* the constructed vectors provided promising results, which could be used to achieve the ultimate goal of a steady supply of artemisinin at affordable prices around the world.

## Introduction

Plants, as green photosynthetic organisms, synthesize and accumulate a diverse array of specialized compounds through secondary metabolic pathways. These compounds (estimated at 200,000) are not normally involved in basic plant growth and development but are essential for plants to survive in response to their environment. Secondary metabolites, on the other hand, are not only important for plant adaptation strategies but also have benefits for humans. For instance, over the last two decades, more than 70% of FDA-approved medicine has been based on secondary metabolites found in plants (Schramek et al., 2010; Yang et al., 2016). *Artemisia annua* L. is a valuable medicinal plant used in Chinese traditional medicine that produces artemisinin, a unique secondary metabolite (Tu, 2011). The impact of artemisinin against *Plasmodium falciparum*, the primary malaria agent, has received considerable attention (Mutabingwa, 2005). Malaria is a deadly disease that kills thousands of people each year and is spread by female Anopheles mosquitos (World Health Organization, 2016). Although artemisinin is widely considered the most effective anti-malarial compound, the low rate of artemisinin production in plants (< 1%) necessitates numerous efforts to improve the artemisinin yield in plants (Ro et al., 2006; Paddon et al., 2013; Shen et al., 2016). Even though semi-synthesis of artemisinin *via* artemisinic acid in engineered yeast was achieved (Turconi et al., 2014), it was unable to meet industrial demands due to significantly higher costs than artemisinin produced in plants (Peplow, 2016). As a result, identifying enzyme-coding genes as well as the regulatory network of transcription factors is critical for transformation strategies and the production of higher yield lines. So, in recent years, there has been a significant amount of research to understand the molecular mechanism underlying the regulation of artemisinin biosynthesis (Hassani et al., 2020; Kayani et al., 2021; Shu et al., 2022).

**Figure 1:**
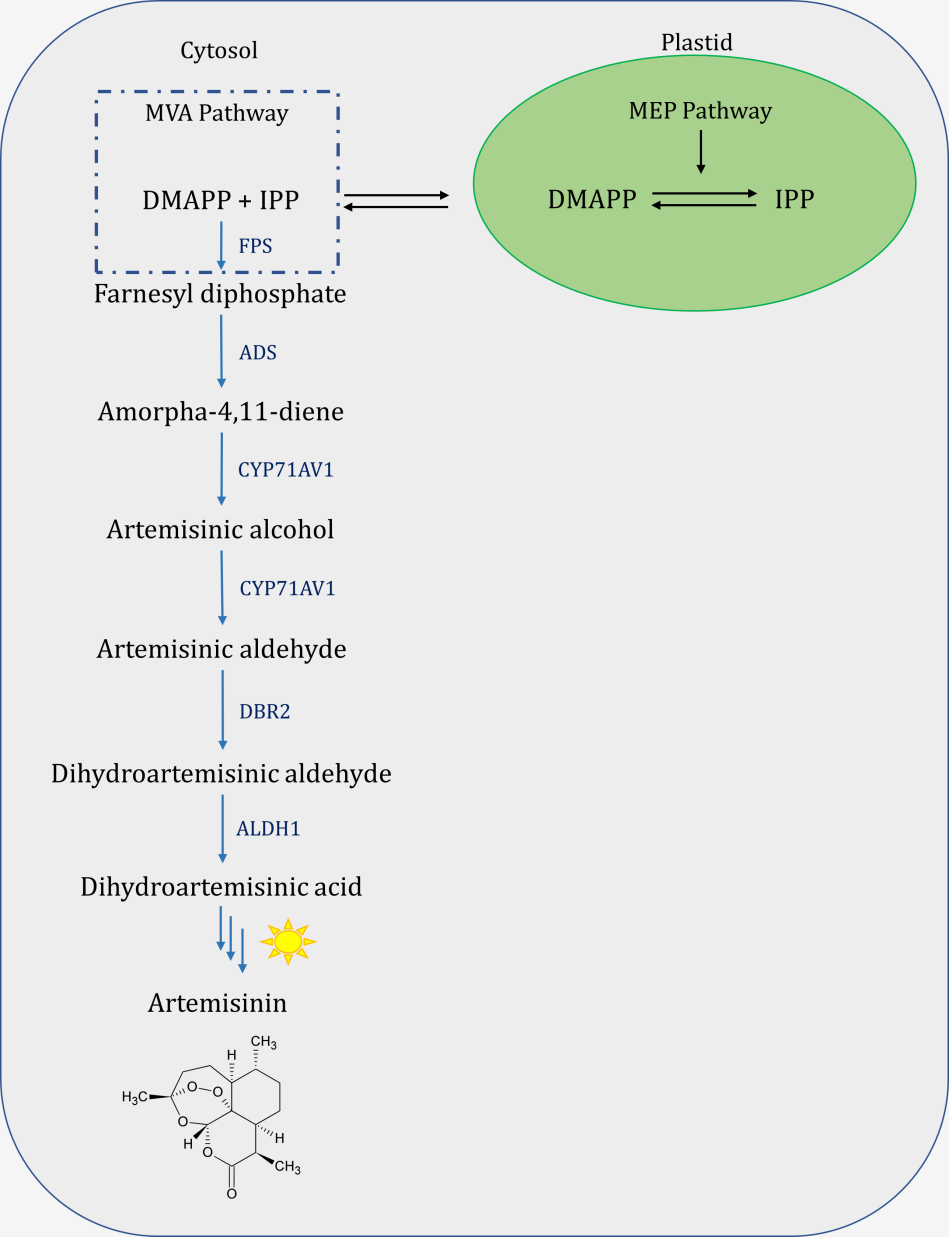
Schematic view of Artemisinin biosynthesis pathway. MVA, mevalonate pathway; MEP, 2-C-methyl-d-erythritol-4-phosphate pathway; DMAPP, dimethylallyl diphosphate; IPP, isopentenyl diphosphate; FPS, farnesyl diphosphate synthase; ADS, amorpha-4,11-diene synthase; CYP71AV1, cytochrome P450 monooxygenase; DBR2, double bond reductase 2; ALDH1, aldehyde dehydrogenase 1.

Artemisinin, like other secondary metabolites, is synthesized by a multi-enzymatic biosynthetic pathway. Terpenoid biosynthesis is initiated by the cytosolic mevalonate pathway (MVA) and plastid methylerythritol phosphate (MEP) derived isopentenyl diphosphate (IPP) and dimethylallyl diphosphate (DMAPP) (Vranová et al., 2013). IPP and DMAPP produced by the MVA pathway are used primarily to power the production of polyisoprenoids, triterpenes, and sesquiterpenes. MEP products, on the other hand, are typically used to drive monoterpene, diterpene, and tetraterpene biosynthesis (Vranová et al., 2013; Yang et al., 2016). However, the biosynthesis of artemisinin is initiated by farnesyl diphosphate synthase (FPS) activity on plastidial and cytosolic derived IPP and DMAPP to convert them to farnesyl diphosphate (FPP) (**Figure 1**) (Schramek et al., 2010). Furthermore, the amorpha-4,11-diene synthase (ADS) forms a double bond or a new ring structure on phosphates, breaking the chemical bond of FPP, generating amorpha-4,11-diene, which is then hydroxylated, and oxidized by cytochrome P450 monooxygenase (CYP71AV1) and cytochrome P450 oxidoreductase (CPR) to artemisinic alcohol, artemisinic aldehyde, and artemisinic acid (Ro et al., 2006; Teoh et al., 2006; Alam et al., 2010). Furthermore, artemisinic aldehyde Δ11(13) reductase (DBR2) reduces artemisinic aldehyde to dihydroartemisinic aldehyde, which is then converted to dihydroartemisinic acid (DHAA) by aldehyde dehydrogenase 1 (ALDH1) as the final compound generated by enzymatic reactions in *A. annua* (Teoh et al., 2006).

Among the various techniques used to boost artemisinin production, including breeding, chemosynthesis, and microbial metabolic engineering (Brisibe and Chukwurah, 2014; Corsello and Garg, 2015), overexpression strategies of the enzyme-coding genes of the artemisinin biosynthetic pathway have proven to be a successful approach. Among the key enzymes well-studied, 3-hydroxy-3-methylglutaryl coenzyme A reductase (HMGR) is an important regulatory enzyme responsible for diverting HMG-CoA into the mevalonate arm of the isoprenoid pathway where it is converted to (IPP) and further to FPP, respectively (Ram et al., 2010). Since FPP is the precursor for essential isoprenoids, including artemisinin, its production by overexpression of farnesyl diphosphate synthase (FPS) has been investigated in *A. annua* (Chen et al., 2000; Han et al., 2006a). DBR2, another essential enzyme in artemisinin biosynthesis, is not only crucial for its catalytic activity but can also be considered as a checkpoint to direct the conversion of artemisinic aldehyde to dihydroartemisinin aldehyde toward artemisinin production (Yuan et al., 2015). Minimizing the activity of competitive branch pathways is a practical strategy for a successful transformation toward a higher yield of the desired product. HMGR, FPS, and DBR2 with checkpoint features could be suitable candidates in this regard.

The biosynthesis of artemisinin in *A. annua* occurs in glandular secretory trichomes (GSTs) of the leaves. GST structures are made up of 10 symmetric cells and are thought to be the main biofactories for artemisinin production, storage, and secretion, however, there is also a report on the production of artemisinin in the non-glandular trichomes of self-pollinated inbred *A. annua* (Judd et al., 2019). The exclusive expression of artemisinin pathway key enzymes in GSTs, nominate them to be the true cell biofactories and the target for metabolic engineering and breeding strategies to improve their efficiency, such as by increasing their density (Mutabingwa, 2005; Schilmiller et al., 2008; Chen et al., 2021). However, the lack of a model plant for GST study, as well as poor knowledge of the mechanism underlying their development, are two limiting constraints. The initiation of GSTs and T-shaped non-secretory trichomes (TSTs) *via* distinct transcription factor (TF) networks, as well as information from key enzyme expression profiles, have led to a possible solution (Ro et al., 2006). Among the most common TF families in *A. annua*, the *AaORA* an APETALA2/ETHYLENE RESPONSIVE FACTOR (AP2/ERF) TF was cloned and found to be trichome specific. Meanwhile, the qPCR analysis showed that its expression pattern decreases throughout leaf development, with the highest expression in young leaves. The HPLC results confirmed the elevation of artemisinin and dihydroartemisinic acid in *AaORA* overexpressed *A.annua* (Lu et al., 2013).

Plant hormones, in addition to transcription factors that regulate gene expression, play an important role in increasing secondary metabolite production (Wasternack and Hause, 2013). Recent studies in *A. annua* have suggested a possible relationship between Jasmonic acid (JA) and the homeodomain-leucine zipper (HD-ZIP) TF, leading to epidermal cell differentiation into glandular trichome (Katsir et al., 2008; Yan et al., 2017). It has been reported that AaTCP15 (teosinte branched1/cycloidea/proliferating) TF responds to both JA and ABA and is required for artemisinin biosynthesis by binding to and activating the promoters of the DBR2 and ALDH1 genes; additionally, AaORA (octadecanoid-derivative responsive AP2-domain protein), a positive regulator of artemisinin biosynthesis, responds to JA and ABA and activates AaTCP15 transcripts. As a result, they collaborate synergistically to activate DBR2, a critical gene for the production of artemisinin, by forming an AaORA-AaTCP15 module (Ma et al., 2021). We have previously demonstrated that AaHD1 interacts with Jasmonate ZIM-domain 8 (AaJAZ8) and suppresses its biological activity, therefore mediating GST initiation and increasing artemisinin content (Yan et al., 2017). There is also research on the role of light in JA-induced artemisinin promotion; binding of AaHY5 to the promoter of AaWRKY9 results in its activation, and AaWRKY9 is a positive regulator of AaDBR2 and AaGSW1 in artemisinin biosynthesis. There is also an interaction between AaWRKY9 and AaJAZ9, which is a repressor in the JA signaling pathway; the absence of JA results in transcriptional repression of AaWRKY9 by AaJAZ9 (Fu et al., 2021).

Based on previous research, we hypothesized that simultaneous cotransformation of GST-specific TFs and artemisinin biosynthetic pathway enzyme-coding genes in *A. annua* would increase the density of the trichome and ultimately the content of artemisinin. So, three independent vectors ProCYP71AV1::*AaORA*, 35S::*HD1* (GST specific TF), and 35S::*EPSPS+HMGR+FPS+DBR2* (artemisinin biosynthetic pathway enzyme-coding genes) were simultaneously co-transferred to *A. annua* to generate the T0 lines. Furthermore, the transformation frequency, regeneration capacity, and artemisinin yield of the transgenic plants of the T1 line were investigated. The results indicated that this simultaneous cotransformation could increase trichome density and the content of artemisinin not only in the T0 line but also in the T1 line.

## Materials and methods

### Plant material

Our laboratory at Shanghai Jiao Tong University grew *Artemisia* annua cultivar ‘Huhao 1’, which originated in Chongqing, PR China. Seed surface sterilization in 75% (v/v) ethanol for 90 seconds was followed by a 15-minute soak in 15% (v/v) NaClO (sodium hypochlorite). To rinse the chemicals, ddH_2_O was used four times. The seeds were then transferred to a sterilized Petri plate containing 0.5% solidified phytagel germination medium MS0 (Murashige and Skoog, 1962) and grown at 25°C with a 16/8 light/dark photoperiod. After four months, the plants were transferred to a larger pot containing a 3:7 mixture of peat soil and vermiculite and relocated to the greenhouse, where they remained at the same temperature and photoperiod.

### Construction of overexpressing vectors

**Figure 2:**
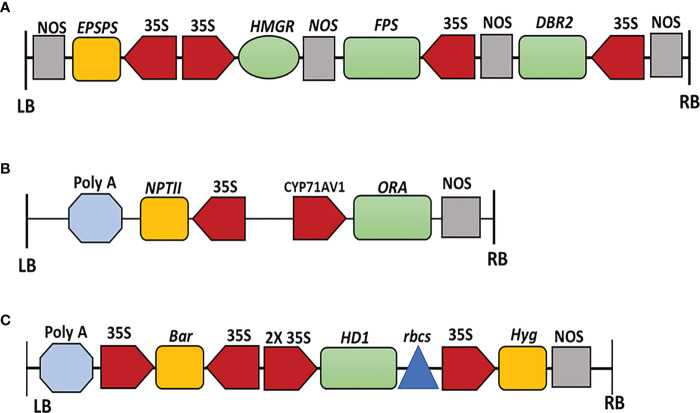
**(A)** Schematic structure of the expression vector pCAMBIA1305-*HMGR-FPS-DBR2-EPSPS* containing *HMGR, FPS, DBR2*, and *EPSPS* genes driven by CaMV35S promoters with *EPSPS* as a selectable marker, respectively. *LB* left border, *RB* right border. **(B)** Schematic structure of the expression vector pCAMBIA2300-*proCYP71AV1-ORA.* The expression of *ORA* is driven by the *CYP71AV1* promoter. The *NPTII* (neomycin phosphotransferase II) is used as the selectable marker. *LB* left border, *RB* right border. **(C)** Schematic structure of the expression vector pHB-*HDG4.* The expression of *HD1* is driven by *2x 35S* promoter with hygromycin B and bar as selectable markers. *LB* left border, *RB* right border. Color code: Terminators, grey; selectable markers, orange; promoters, red; genes, green; Poly A tail, light blue; *rbcs*, dark blue.

We had previously amplified the *AaHMGR* (AF142473), *AaFPS* (AF112881), and *AaDBR2* (PWA95605.1) Open Reading Frames (Wang et al., 2011; Shen et al., 2018). PCR products were separated on 1% Agarose gel electrophoresis and purified using the AxyPrep DNA Gel Extraction Kit (Axygen Biosciences, USA). Purified PCR products were then cloned into the pJET2.1 vector (Promega), transformed into DH5α high-efficiency competent cells, and grown on LB medium supplemented with Carbenicillin (Cb). Three positive colonies were subjected to colony PCR and submitted for sequence analysis (Sangon sequencing company, Shanghai). For sequence alignment, the DNAMAN software (version 5.6) was utilized. EPSPS was substituted for the hygromycin gene in the *XhoI* restriction site of pCAMBIA1305. The vector pCAMBIA1305.1-EPSPS-HMGR was created by inserting 35S-HMGR-nos into the *HindIII* and *EcoRI* restriction sites of pCAMBIA1305-EPSPS using 5× In-fusion HD Enzyme Primix (Clontech, Dalian, China). The *FPS*-nos-35S-*DBR2* fragment was then inserted into the *NcoI* and *BstEII* restriction sites of pCAMBIA1305.1-EPSPS-*HMGR* (**Figure 2A**).

The full-length CDS for *ORA* (JQ797708) was amplified and digested with *BamHI* and *SacI*. The full-length ORF was then cloned into the *BamHI* and *SacI* sites of the pCAMBIA2300+ vector under the CYP71AV1 promoter to create pCAMBIA2300-proCYP71AV1::*AaORA*::NOS (**Figure 2B**).

The ORF of *AaHD1* (KU744599), was amplified and cloned into the PHB vector (**Figure 2C**). After validating the transformation, the positive colonies were transferred into *Agrobacterium tumefaciens* strain EHA105 and inoculated into *A. annua.*

### Agrobacterium-mediated transformation and T0 generation

To generate T0 transgenic plants, after 3-4 months seedlings with a length of 4-5 cm were cut and inoculated for three days at 25°C with *Agrobacterium tumefaciens* carrying constructed vectors with selectable markers (McCormick et al., 1986). The inoculated cuttings were moved to MS1 medium (MS + 0.3 mg/L naphthalene-1-acetic acid (NAA) + 2.5 mg/L 6-benzyl adenine (6-BA) + 250 mg/L carbenicillin + 50 mg/L kanamycin), and then to rooting medium (250 mg/L carbenicillin + half-strength MS0). The resistant seedlings and wild types were then transplanted to the pots with the known compositions ((EC (dS/m) 0.14, pH 7.32, Avail. K (ppm) 181.7, Avail. P (ppm) 181.7, Avail. N (ppm) 111.6, NH_4_^+^ (ppm) 7.86, NO_3_^-^ (ppm) 2.67, CEC (cmol(+)/kg) 306.8, total K (ppm) 2063, total N (%) 0.19, and total C (%) 1.92) and sand (autoclaved) in a 3:1 (w/w) ratio and grew at 25°C with 16/8 light/dark photoperiod.

### DNA extraction and PCR analysis

Using liquid nitrogen, fresh young leaves were ground to powder, and genomic DNA was extracted using the cetyltrimethylammonium bromide method (Fütterer et al., 1995). The forward primer for *HMGR*, *FPS*, *DBR2*, and *HD1* was chosen from the *EPSP* marker gene. Because *AaORA* was driven by the CYP71AV1 promoter, the forward primer was designed within its sequence, and the reverse primer was selected from the CDS of *AaORA.* **Table 1** shows a list of primers. The PCR reaction was carried out in a 20 μL tube using the LA TaqR Kit (Takara). DNA denaturation was set at 94° C for 3 minutes, followed by 30 cycles of 94° C for 30 seconds 57° C for 30 seconds, and 60 seconds at 72°C, and a final extension of five minutes at 72° C. For product determination, 1.0% agarose gel electrophoresis was performed.

**Table 1:** List of the primers used in this experiment.

Gene ID	Sequence (5’-3’)	Purpose
*HMGR*-FP	ATGGATCTCCGTCGTAAACTGCC	PCR
*HMGR*-RP	TCACACCTTTGACGCAATTGCTG	PCR
*FPS-*FP	ATGAGTAGCATCGATCTGAAAT	PCR
*FPS-*RP	CTACTTTTGCCTCTTGTAGATT	PCR
*DBR2*-FP	ATGTCTGAAAAACCAACCTTGT	PCR
*DBR2*-RP	CTAGAGGAGTGACCCTTTGTCA	PCR
*ORA*-FP	ATGTTTGCTACTTGCATTCGCAC	PCR
*ORA*-RP	AGTCATCATCTGTCAATGTATCC	PCR
*HD1*-FP	ATGTATGGAGATTGTCAAGTGA	PCR
*HD1-*RP	TTAACCTTGGGAAGTCGGGCTT	PCR
CaMV 35S	GACGCACAATCCCACTATCC	PCR
*EPSPS-*XhoI-FP	CGGGATCTGCGAAAGCTCGAGATGGCGCAAGTTAGCAGAAT	Vector construction
*EPSPS*-XhoI-RP	CTGTCGATCGACAAGCTCGAGTTAGTCGTTAAGGTGAACTC	Vector construction
*HMGR*- HindIII-FP	CGACGGCCAGTGCCAAGCTTGCATGCCTGCAGGT	Vector construction
*HMGR*- EcoRI-RP	ATGACCATGATTACGAATTCCCGATCTAGTAACA	Vector construction
*FPS*- NcoI-FP	CGGGGGACTCTTGACCATGAGTAGTACCGATCTGAA	Vector construction
*DBR2*-BstEII-RP	GAAATTCGAGCTGGTCACCCTAGAGGAGTGACCCTTTGT	Vector construction
*HD1*-BamH1-FP	GGATCCATGTATGGAGATTGTCAAGTGATGTC	Vector construction
*HD1*-Spe1-RP	ACTAGTACCTTGGGAAGTCGGGCT	Vector construction
*ORA*-BamHI-FP	CCGGATCCATGTTTGCTACTTGCAT TCGCAC	Vector construction
*ORA*-SacI-RP	CCGAGCTCTCAAAAAAAAAAAAAGTCATCATCTG	Vector construction
*actin*-FP	CCAGGCTGTTCAGTCTCTGTAT	qRT-PCR
*actin*-RP	CGCTCGGTAAGGATCTTCATCA	qRT-PCR
*HMGR-*qRT*-*FP	AGCAGATTTGCTAGGCTCCA	qRT-PCR
*HMGR-*qRT*-*RP	TGGACACCCTTTGACACCAT	qRT-PCR
*FPS-*qRT-FP	TCTGCCCTTGGTTGGTGTAT	qRT-PCR
*FPS-*qRT*-*RP	AATTCCATCGTTCGCAGCAA	qRT-PCR
*DBR2-*qRT*-*FP	TCTGCCTACAAGATGGGCAA	qRT-PCR
*DBR2-*qRT*-*FP	CCGGCAGAAGAAGGAGAGAT	qRT-PCR
*ORA-*qRT-FP	TGAACAAGCTGCATTGGCTT	qRT-PCR
*ORA-*qRT-RP	GGACCGATCATCACAGCCTA	qRT-PCR
*HD1-*qRT-FP	TGCATTGCTCTCCTTCCTCA	qRT-PCR
*HD1-*qRT*-*RP	GGTTGAGTTTGGCTGTTGGT	qRT-PCR

### RNA isolation and gene expression analysis

The initial leaves of three-month-old transgenic and control plants were collected, transferred to RNase-free tubes, and ground to powder in liquid nitrogen. The RNeasy Plant Mini Kit (Qiagen, Germany) was used to extract total RNA from the samples. DNase treatment was carried out using DNase I Kit (Takara, Japan) before reverse transcription, and cDNA was reverse transcribed using an RT kit (Promega, USA). The extracted RNA was quantified using a Nanodrop-2000 spectrophotometer (Thermo Fisher Scientific, USA). qRT-PCR was performed using the 7500 Real-Time PCR system (Applied Biosystems, USA) with Fast Start Universal SYBR Green Master Mix (Roche Diagnostics, Germany) as previously described (Fu et al., 2016). qRT-PCR was performed in triplicate and the relative expression level was calculated using the 2^-ΔΔCt^ method (Livak and Schmittgen, 2001).

### HPLC analysis and artemisinin content measurements

To determine the artemisinin content, the leaves of experimental plants were collected and dried at 50°C for 48 hours before being powdered with a mortar and pestle. 0.1 g/sample was vigorously mixed twice with 2 ml of methanol and disrupted by an ultrasonic processor at 55Hz for 30 minutes (Shanghai Zhisun Instrument Co. Ltd model JYD-650) before centrifugation for 10 min at 12000 rpm. The supernatant was transferred to a new 2 ml tube and filtered using a Sartorious ^®^ 0.25 m nitrocellulose 0.25 μm pore size membrane. 200 μL filtrate was injected into the HPLC system (Waters Alliance 2695, Milford, MA, USA) coupled with a Waters 2420 ELSD detector (Zhang et al., 2009; Lu et al., 2013). Artemisinin standards were purchased from Sigma (Sigma-Aldrich, St. Louis, MO, USA), and each sample was run in triplicate.

### T1 progeny generation

Based on HPLC data from the T0, two transgenic lines (E and F) with the highest artemisinin content were chosen for the development of T1 progeny. To prevent cross-pollination, the T0 plants were covered with paper bags. The seeds from the T0 lines were harvested and stored in the warehouse of the school of agriculture and biology, Shanghai Jiao Tong University. During the spring of 2019, the seeds were cultivated in a 50:50 peat-lite/sand medium and kept in the germination room at 25°C with 16/8 hours of light/dark to obtain T1 transgenic progeny plants. Each line produced a total of 24 independent T1 plants, which were then subjected to DNA extraction and PCR analysis to confirm the presence of the inserted genes. Positives were transferred to the field and grown for further research.

### GSTs density counting

To assess GST density, mature leaves (leaf 9) from transgenic plants T0 and T1 with increased artemisinin content and wild-type *A. annua* were collected. Images from the adaxial side of the leaves were taken using fluorescence microscopy (Olympus BX51, Tokyo, Japan) equipped with an A×5 objective lens. The fluorescence excitation was captured at 450–480 nm, and the ImageJ software (http://rsb.info.nih.gov/ij) was used to measure the leaf area and count the number of GSTs, as previously described (Cheng et al., 2014).

### Statistical analysis

All parameters were expressed as the mean standard deviation of three replicates. All data came from three biological repeats with three technical replicates. The Student’s t-test was used to analyze significant differences between mean values of treatments using one-way ANOVA.

## Results

### PCR and qRT-PCR analysis of T0 transgenic plants

**Figure 3:**
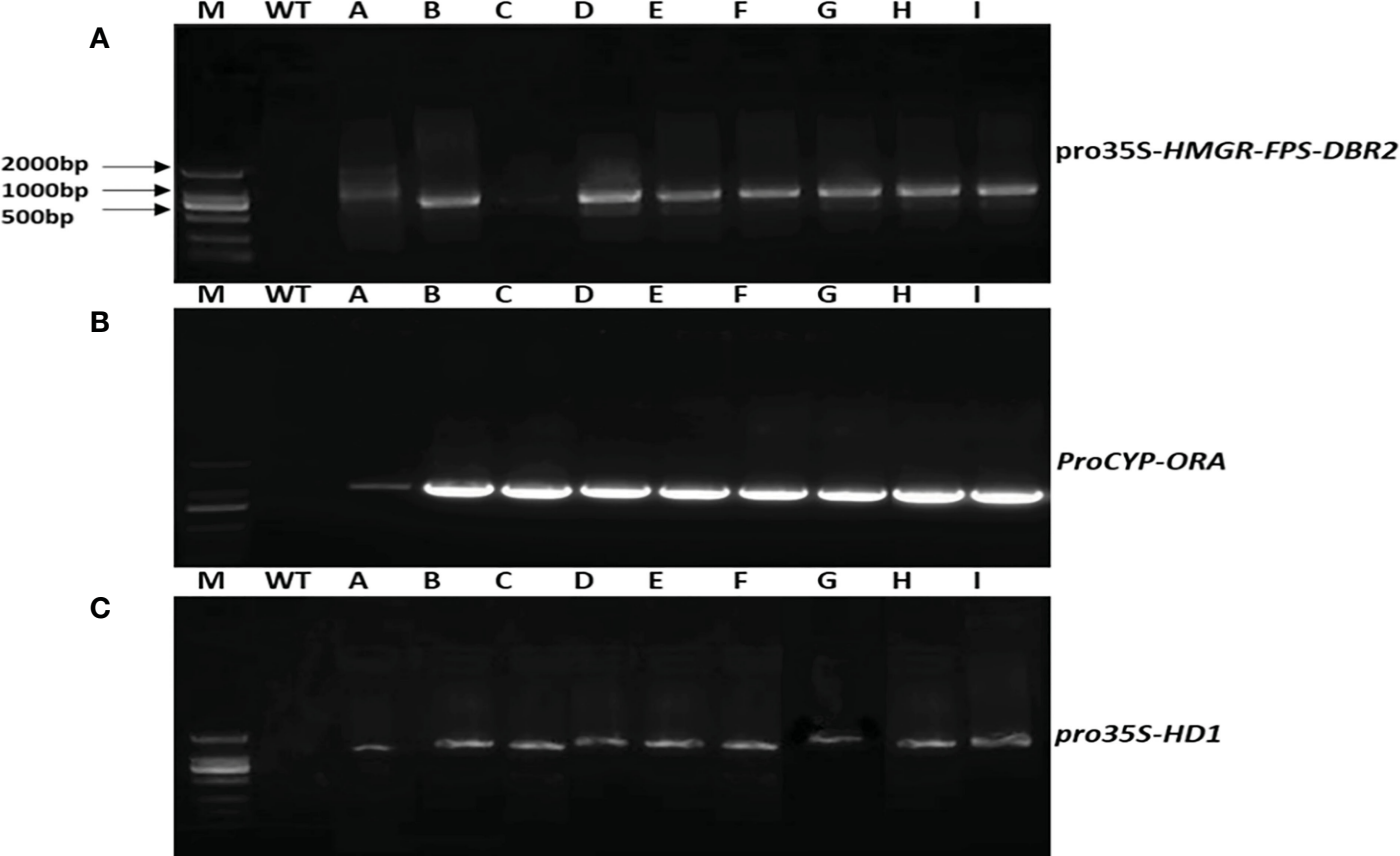
Gel electrophoresis illustration of PCR products from transgenic *A annua* T0 lines. **(A)** PCR products for *pro35S-HMGR-FPS-DBR2* using *EPSP* primers as a selectable marker (800bp). **(B)** PCR products for *proCYP-ORA*, using a forward primer from *CYP71AV1* promoter and reverse primer from CDS or *AaORA* (1160bp). **(C)** PCR products for *pro35S-HD1*, using a forward primer from EPSPS as the selectable marker and reverse primer from CDS or *AaHD1* (1600bp).

We were able to extract and clone the *HMGR, FPS, DBR2, HD1*, and *ORA* target genes using PCR. *Agrobacterium*-mediated transformation was performed using the above-mentioned overexpression vectors and transgenic plants were generated, which were then assessed for the positive integration of the target genes in their genomes, using appropriate pairs of primers (**Table 1**) and their DNA as the template. **Figure 3** shows the results of PCR analysis of the DNA extracted from T0 transgenic plants.

**Figure 4:**
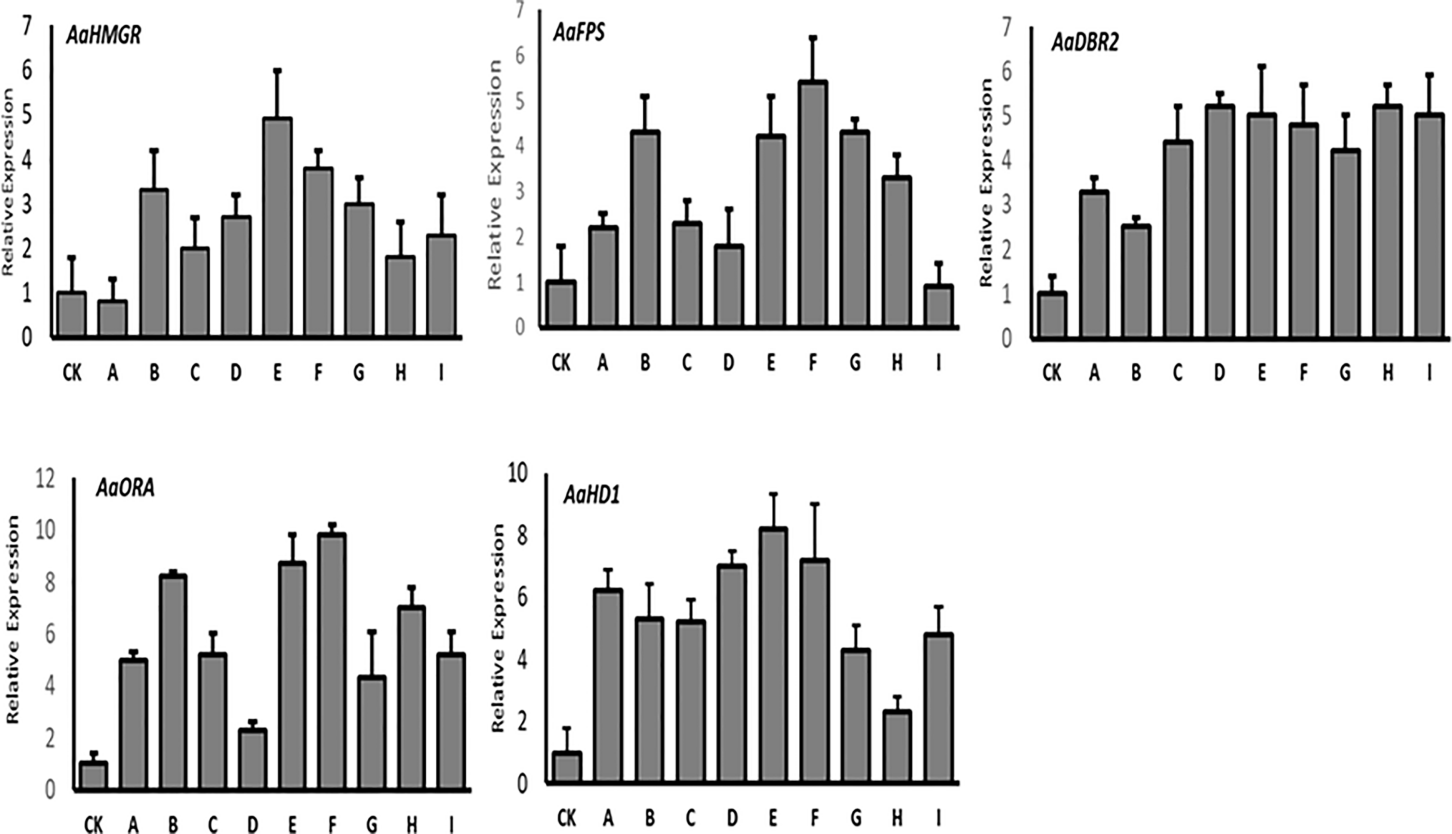
The qRT-PCR analysis of the expression levels of *HMGR, FPS, DBR2, ORA*, and *HD1* in T0 transgenic *A. annua*. Actin was used as internal reference. The X axis represents the relative gene expression level, and the Y axis represents the different plant lines. CK, wild type. The analysis is performed in technical triplicates.

The qRT-PCR results of the T0 lines showed that the first three enzyme-coding genes (*HMGR, FPS*, and *DBR2*) were expressed at varying levels. Except for line “A”, the *AaHMGR* expression levels were all increased, with the line “E” being 4.9-fold higher than the control. For *FPS*, the highest expression levels were observed in lines “F” and “B”, which were 5.4- and 4.3-fold higher than the control, respectively. However, the *AaFPS* expression in line “I” was lower than the control. In the case of *DBR2*, all transgenic lines showed increased gene expression, with line “D” showing the highest increase (5.2-fold) over control and line “B” showing the lowest increase (2.8-fold). Two trichome-specific transcription factors (*AaORA* and *AaHD1*) were found to be highly expressed in all lines, with a significant level of *AaORA* in line “F” (9.8-fold) and *AaHD1* (8.2-fold) in line “E” (**Figure 4**). These findings supported the gel electrophoresis of our T0 transgenic lines and demonstrated that target genes integrated into genomes are successfully expressed.

### Trichome density and artemisinin measurement in T0 transgenic lines

**Figure 5:**
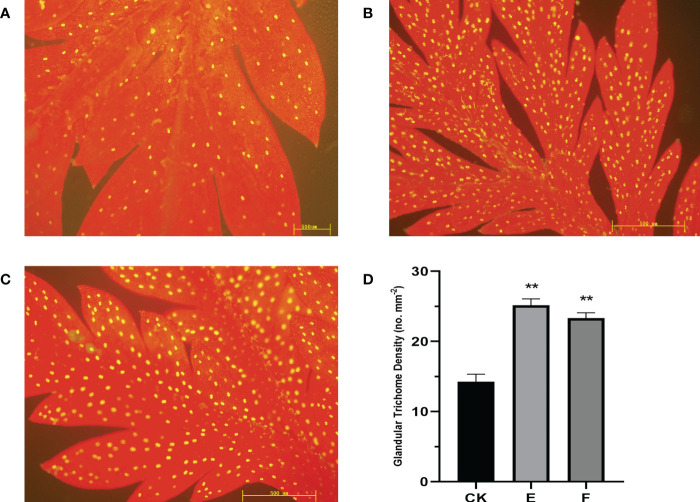
The expression of *AaORA* and *AaHD1* affects the glandular trichome initiation and density in T0 transgenic *A annua*. **(A)** The glandular trichomes on the adaxial side of mature leaves derived from Wild type (CK) *A annua* plants. **(B)** The glandular trichomes on the adaxial side of mature leaves derived from the Overexpressed T0 line E *A annua* plants. **(C)** The glandular trichomes on the adaxial side of mature leaves derived from the overexpressed T0 line F *A annua* plants. **(D)** Glandular trichomes density of mature leaves derived from CK and T0 transgenic E and F lines. The X and Y axis represent glandular trichome density and different plant lines, respectively. Data are given as means ± SD (n = 3) (*, P < 0.05; **, P < 0.01 Student’s t-test). Fluorescence microscopy was used to observe the images by capturing the red autofluorescence of chlorophyll and the yellow autofluorescence of the glandular trichome.

Based on the results of the gene expression, lines “E” and “F” which had higher expression levels are represented here. The density of trichomes in overexpression “E” and “F” lines was investigated using three independent repeats for each line. Compared to the control, GSTs on the adaxial side of mature leaves increased by up to 76.4% in Line E and 63.5% in Line F (**Figure 5**).

**Figure 6:**
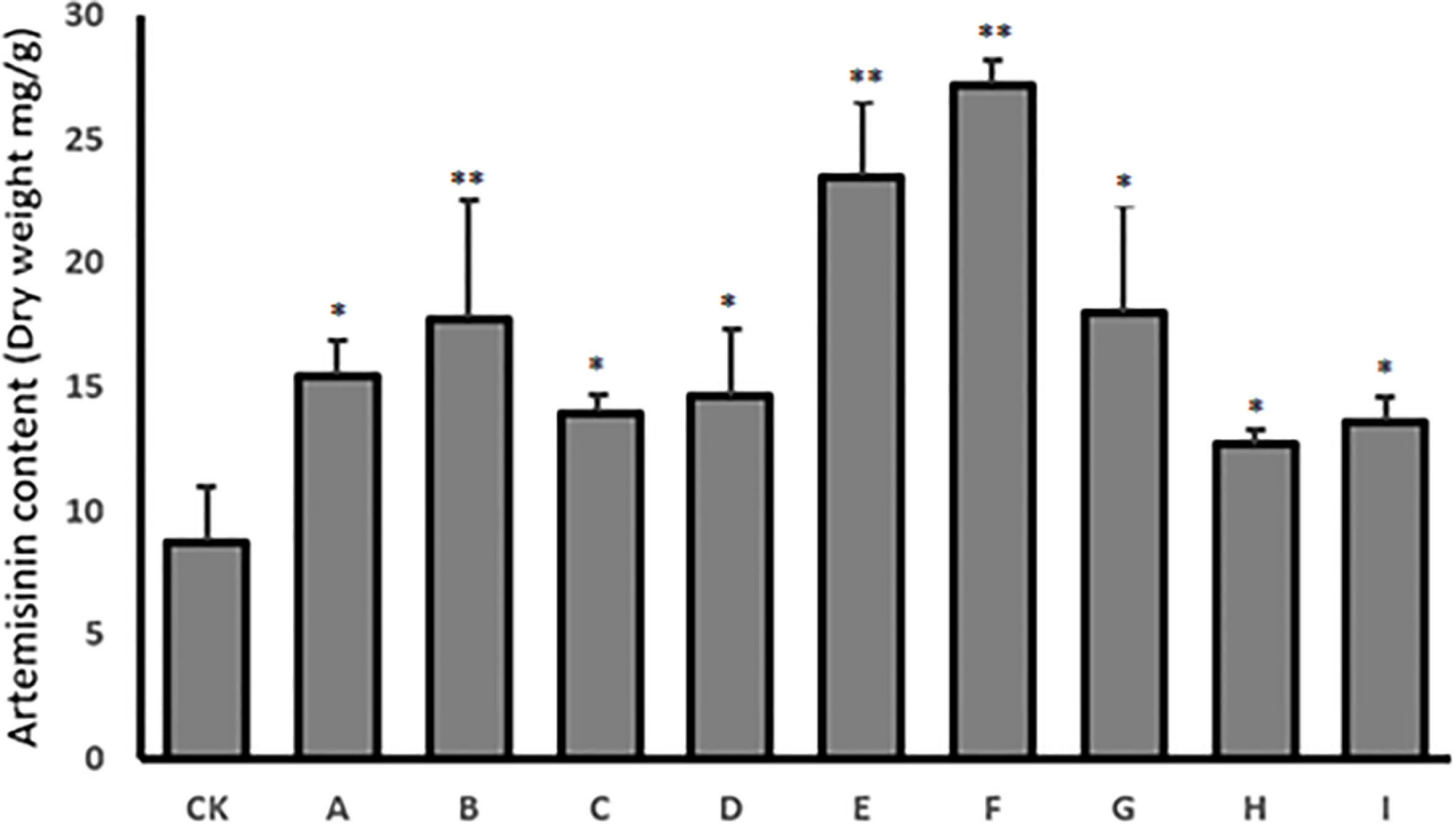
HPLC analysis of artemisinin content in nine T0 transgenic lines (A–I) and the wild type plant as control (CK). Data are given as means ± SD (n = 3) (*, P < 0.05; **, P < 0.01 Student’s t-test).

HPLC was used to quantify the content of artemisinin in nine transgenic lines and the control, to confirm the effectiveness of co-transformation in increasing artemisinin content in transgenic T0 lines. The results showed that the artemisinin content had increased in all nine transgenic plants. The maximum increase in artemisinin level was 23.5 mg g^−1^ (2.35%) dry leaf weight in transgenic line E and 27.2 mg g^−1^ (2.72%) dry leaf weight in transgenic line F, which were 2.8- and 3.2-fold higher than in control plants (8.5 mg g^−1^, 0.85%), respectively (**Figure 6**).

### PCR analysis of T1 transgenic plants

**Figure 7:**
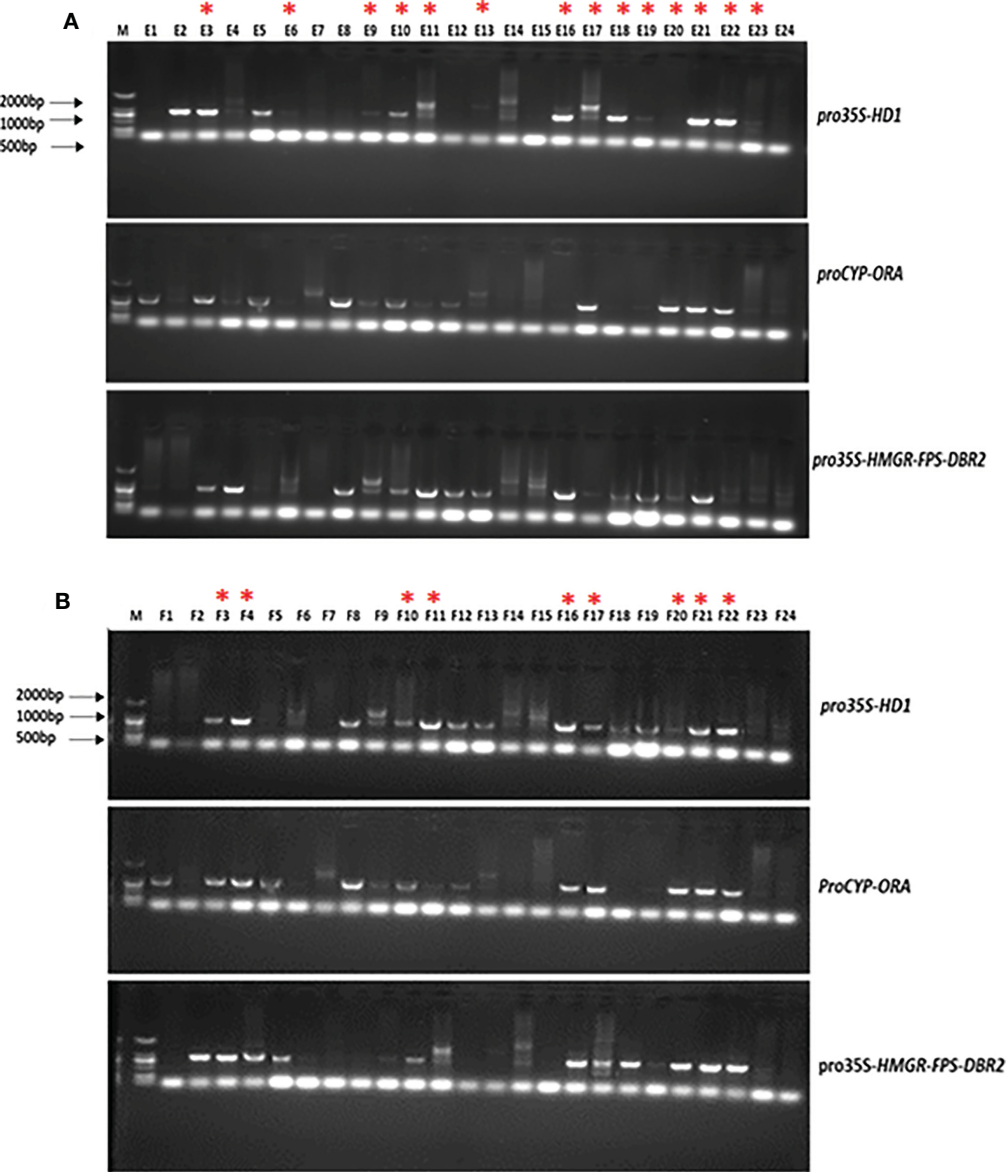
Gel electrophoresis illustration of PCR products from high artemisinin content T1 transgenic lines. **(A)** 24 plants from Line (E, **B**) 24 plants from Line (F) The selected lines are denoted by a red asterisk.

PCR was performed with extracted DNA from lines E1-24 and F1-24, to confirm the positive integration of the target genes in their genome, using appropriate pairs of primers. The results of the PCR analysis of the DNA extracted from T1 transgenic plants are presented in **Figure 7**. The lines showed positive integration of target genes in their genome were chosen for further analysis based on the electrophoresis results. The selected lines are denoted by an asterisk (**Figure 7**).

### qRT-PCR analysis of T1 transgenic plants

**Figure 8:**
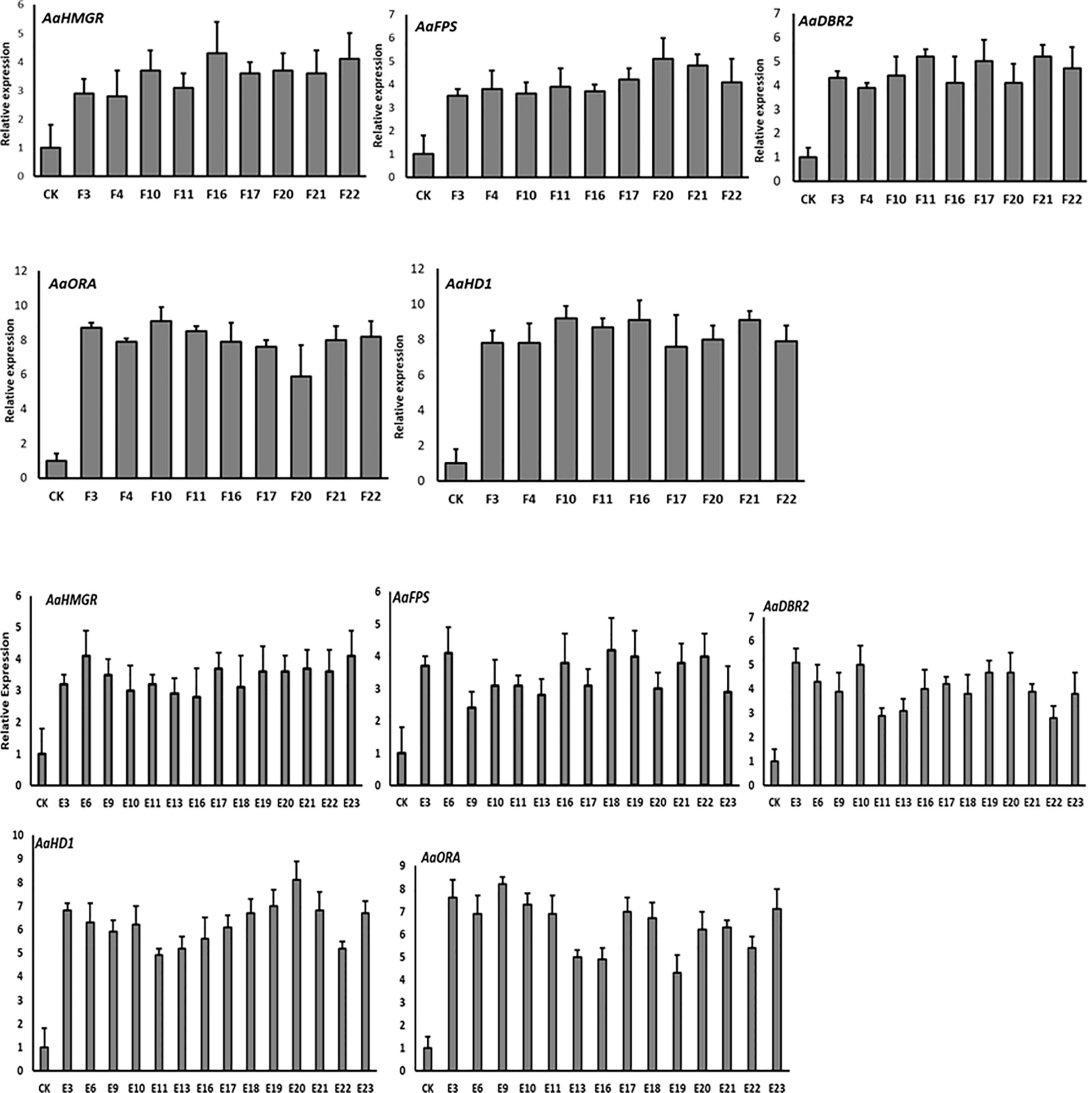
qPCR analysis of target genes in T1 selected lines.

The results of the qPCR analysis of T1 progeny plants revealed that the plants that had integrated all 5 genes into their genome showed an overexpression pattern of their target genes (**Figure 8**). The results were consistent with the results of the PCR analysis.

### Measurement of trichome density in T1 transgenic plants

**Figure 9:**
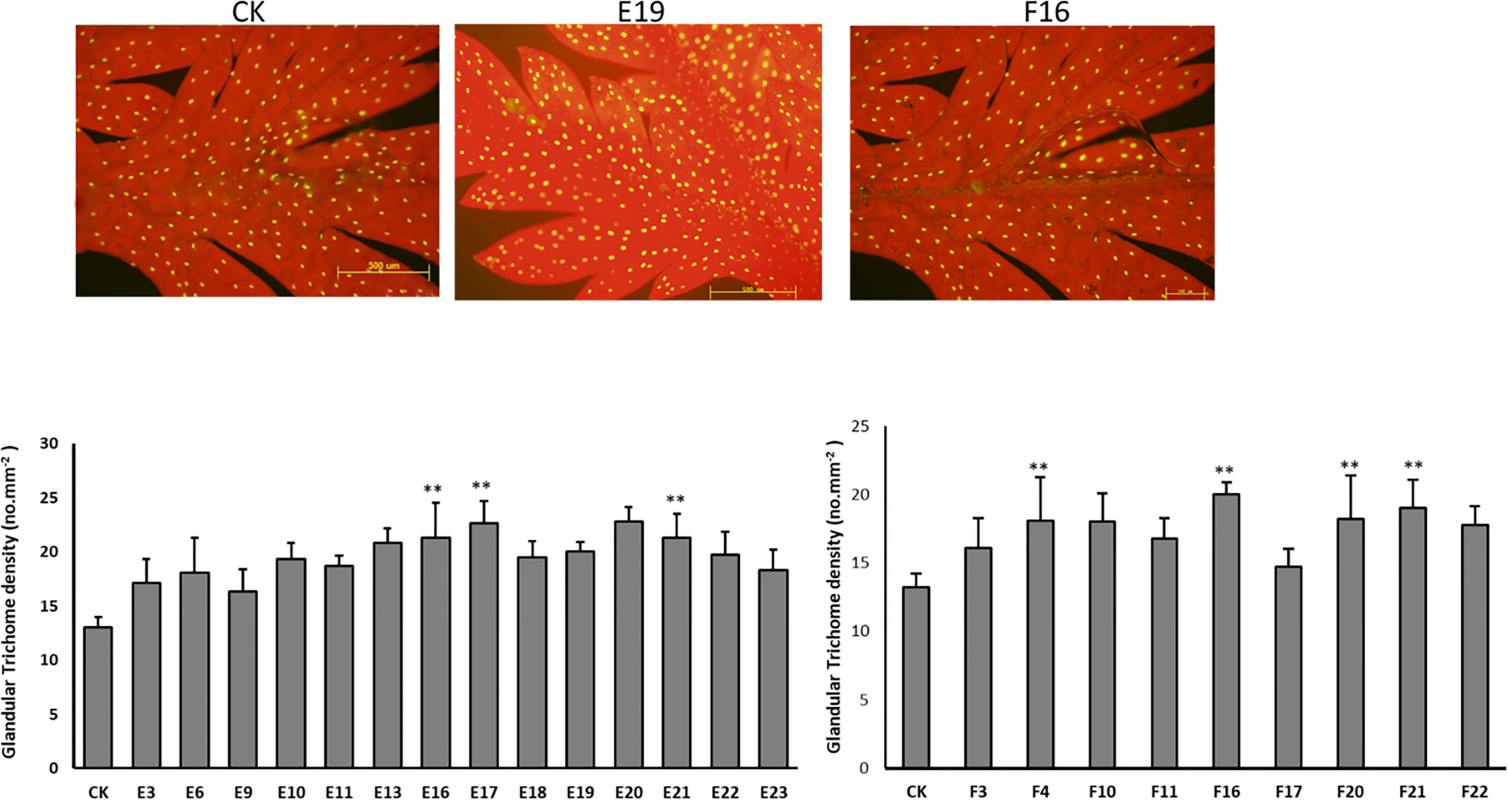
The density of glandular trichomes in mature leaves derived from CK and T1 transgenic plants. Data are given as means ± SD (n = 3) (**, P < 0.01 Student’s t-test).

Following the PCR and qPCR results, the selected plants were examined for trichome density (**Figure 9**). Transgenic plants, as expected, have a greater number of trichomes on their leaf surface. Significant results were obtained from lines 16, 17, and 21 of line E, as well as lines 4, 16, 20, and 21 of line F.

### Analysis of artemisinin content by HPLC in T1 transgenic lines

**Figure 10:**
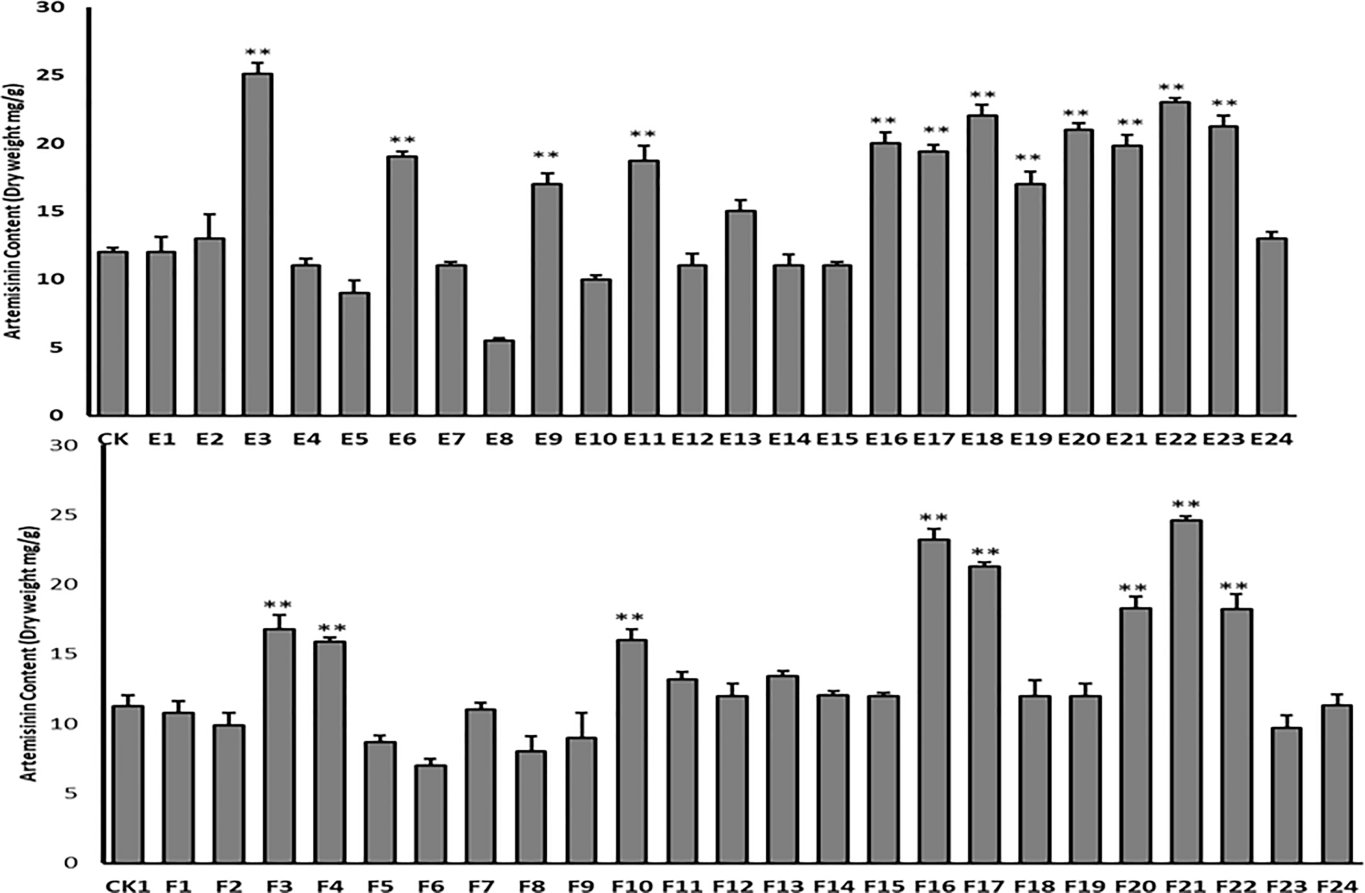
HPLC analysis of artemisinin content in T1 transgenic lines (E1-24 and F1-24) and wild-type plant as control (CK). Data are given as means ± SD (n = 3) (*, P < 0.05; **, P < 0.01 Student’s t-test).

HPLC analysis was performed to confirm the successful integration of target genes into the genome of the T1 progeny transgenic plant. HPLC analysis revealed an increase in artemisinin content in lines E and F (**Figure 10**). The highest artemisinin content was measured in lines E3 (25.1 mg g^-1^ (2.51%) leaf dry weight) and F21 (24.6 mg g^-1^ (2.46%) leaf dry weight). These findings were supported by qPCR and trichome density.

## Discussion

(Willcox, 2009; Peplow, 2016)

In this study, we overexpressed the artemisinin mainstream enzyme-coding genes to reduce substrate flux to side branches and increase artemisinin production. To gain this, we isolated, cloned, and over-expressed *HMGR*, which correlates the carbon flux from the cytosolic MVA pathway to the artemisinin pathway, *FPS*, which catalyzes the initial step of essential isoprenoid synthesis, and *DBR2*, which is a checkpoint for directing the conversion of artemisinic aldehyde to dihydroartemisinic aldehyde toward artemisinin production (Han et al., 2006b; Ram et al., 2010; Ikram et al., 2019). The high expression of *HMGR, FPS*, and *DBR2* in T0 transgenic plants was followed by higher artemisinin content (2.8- and 3.2-fold higher artemisinin) in Line E and F, respectively; indicating that these genes are suitable candidates for overexpression strategies and useful for increasing artemisinin content in *A. annua*. Several studies have shown that individually transforming enzyme-coding genes or TFs can boost the production of target metabolites in plants (Aquil et al., 2009; Yuan et al., 2015; Shi et al., 2017; He et al., 2022). Over-expression of *DBR2* (Yuan et al., 2015), *HMGR* (Aquil et al., 2009), and *FPS* (Banyai et al., 2010), for example, increased artemisinin content by up to 2, 2.2, and 2.5 folds, respectively. Another study found that overexpressing *HMGR* from *Catharanthus roseus* in *A. annua via Agrobacterium* resulted in a nearly 50% increase in artemisinin content when compared to non-transgenic plants (Nafis et al., 2011). Also, *AaWRKY* 1 overexpression in *A. annua* resulted in *CYP71AV1* upregulation, which increased artemisinin content by up to 1.8 times higher than wild-type *A. annua* (Han et al., 2014).

To increase GST density in transgenic plants, we also overexpressed two important trichome-specific TFs (Lu et al., 2013; Yan et al., 2017). The over-expressing pCAMBIA2300-proCYP71AV1::*AaORA* and pHB::*AaHD1* vectors were constructed and transformed into the plants. The higher artemisinin content in the T0 and T1 transgenic lines was associated with a higher expression level of these genes as well as an increased number of GSTs. Previous studies indicated that overexpression of *AaORA* and *AaHD1* (trichome-specific TFs) can increase trichome density in transgenic lines, thus increasing the artemisinin content by up to 1.5 folds (Lu et al., 2013; Yan et al., 2017). Overexpression of AaGSW2, a specific WRKY transcription factor for trichomes, has also been reported in trichomes, resulting in increased GST density in *A. annua*; however, knockdown lines showed impaired GST initiation. They also discovered that the ectopic expression of the *Mentha spicata* and *Mentha haplocalyx AaGSW2* homologs in *A. annua* resulted in GST formation (Xie et al., 2021). *AaMYB17* and *AaMIXTA1* are also reported to positively regulate glandular trichome formation in *A. annua* and overexpressing lines demonstrated increased glandular initiation, whereas repression of these TFs resulted in decreased glandular trichome density (Shi et al., 2018; Qin et al., 2021). Also, *AaMIXTA1* and *AaHD8* form a regulatory complex that positively regulates trichome initiation and cuticle development in *A. annua*, ultimately increasing the expression of *AaHD1* and cuticle development genes (Yan et al., 2018).

Our findings from the T0 and T1 transgenic lines suggested that the co-transformation of these genes could be an efficient strategy to increase artemisinin content while also forming a stable transformation in progeny plants.

## Conclusion

Since the WHO recommended artemisinin combination therapy as the first line of treatment in the battle against malaria, there has been an increase in demand for its supply, but there is still a significant shortfall to meet global demand. Although semisynthesis artemisinin production from genetically modified yeast is conceivable but more expensive than production from plants, *A. annua* remains the primary commercial outlet for artemisinin. Therefore, transformation strategies will continue to be the primary approach to meet market expectations. We demonstrated in this study that the simultaneous co-transformation of three mainstream enzyme-coding genes and two trichome-specific TFs can promote artemisinin content in *Artemisia annua* to generate a high artemisinin-yielding progeny plant, which may secure a steady supply of artemisinin globally at affordable prices.

## Data availability statement

The raw data supporting the conclusions of this article will be made available by the authors, without undue reservation.

## Author contributions

DH designed and performed the experiments and drafted the manuscript. AT evaluated and analyzed the data, prepared figures, and revised the manuscript. XF provided the overexpression vectors and revised the manuscript. WQ, LH, and YM revised the manuscript. KT served as the principal investigator and corresponding author of this research. All authors contributed to the article and approved the submitted version.
